# Identifying Gaps in Caries Prevention and Management: A Multi-Institutional Mixed-Methods Study

**DOI:** 10.21203/rs.3.rs-7963779/v1

**Published:** 2025-12-22

**Authors:** Suhasini Bangar, Janelle Urata, Oluwabunmi Tokede, Sayali Tungare, Heather Weidner, Aaron Truong, Urvi Mehta, Alfa-Ibrahim Yansane, Gregory Olson, Donald Worley, Emily W. Sedlock, Joanna Mullins, Ryan Brandon, D. Brad Rindal, Todd Johnson, Krishna Kumar Kookal, Nicholas Skourtes, Swaroop Gantela, Heiko Spallek, Joel White, Elsbeth Kalenderian, Muhammad Walji, Ana Neumann

**Affiliations:** The University of Texas Health Science Center McWilliams School of Biomedical Informatics; University of California San Francisco; The University of Texas Health Science Center at Houston School of Dentistry; The University of Texas Health Science Center McWilliams School of Biomedical Informatics; HealthPartners Institute; Willamette Dental; University of Alabama at Birmingham, School of Dentistry,; University of California, San Francisco; The University of Texas Health Science Center at Houston School of Dentistry; The University of Texas Health Science Center at Houston School of Dentistry; UTHealth Houston School of Dentistry; Willamette Dental; Willamette Dental; HealthPartners Institute; The University of Texas Health Science Center McWilliams School of Biomedical Informatics; The University of Texas Health Science Center McWilliams School of Biomedical Informatics; Willamette Dental; University of Sydney; The University of Sydney, School of Dentistry; University of California San Francisco; University of Pretoria; UTHealth Houston School of Dentistry; The University of Texas Health Science Center at Houston School of Dentistry

**Keywords:** caries detection/diagnosis/prevention, electronic dental records, caries, dental informatics/bioinformatics, dental public health

## Abstract

**Background:**

Despite established evidence-based guidelines, the prevention and management of dental caries varies across clinical settings. This study aimed to identify and understand quality gaps in caries prevention and management across four dental institutions.

**Methods:**

A mixed-methods study was conducted across four large dental institutions. Three data sources were integrated: (1) structured chart reviews (n = 2,000) using six validated dental quality measures; (2) semi-structured interviews (n = 102) with patients, caregivers, staff, and dental providers; and (3) ethnographic observations of clinical care (n = 64) using the AEIOU framework. Data were triangulated using thematic coding and root cause analysis. Quality gaps were categorized as quantitative and qualitative gaps, and system-level challenges.

**Results:**

Three categories of gaps emerged. (1) Quantitative gaps included low sealant placement (33.7%–54.6% in ages 6–9; 17.5%–43.0% in ages 10–14), inconsistent caries risk documentation (50.4%–99.6%), and high rates of untreated (23.1%–56.4%) and no new decay rates (65.9%–80.6%). (2) Qualitative gaps included limited documentation of preventive therapies, oral hygiene instruction, and nutritional counseling. Providers cited time constraints, unclear CRA protocols, and EHR usability issues. Interviews revealed unclear team roles and limited patient awareness of preventive options. (3) System-level challenges included workflow inefficiencies, fragmented responsibilities, and poor integration of CRA and preventive codes into clinical routines. Observations confirmed misalignments between documented and delivered care and missed opportunities for risk communication and same-day preventive interventions.

**Conclusions:**

Substantial quality gaps persist in caries prevention and management despite institutional protocols. These gaps result from a complex interplay of systems, providers, and patient-level factors. Findings emphasize the need for coordinated data-informed strategies to improve the consistency and effectiveness of preventive care. This study highlights the value of integrating multiple data sources and user-centered methods to inform quality improvement in dental settings.

## INTRODUCTION

Dental caries remains one of the most prevalent chronic conditions in the United States, affecting more than one in five adults with disproportionate impacts on low-income and underserved populations (1). Despite significant progress in developing and implementing preventive strategies, clinical guidelines, and evidence-based interventions, caries management in everyday practice underperforms the established standards of care (2, 3). These gaps between recommended best practices and daily clinical practice contribute to persistent oral health disparities.

Advancements in dental informatics and the widespread adoption of electronic health records (EHRs) have enabled the use of longitudinal clinical data to assess and improve the quality of oral health care through dental quality measures (DQMs). These measures aim to promote evidence-based, informed, patient-centered care (4). Our prior research has demonstrated the feasibility and validity of implementing DQMs to evaluate performance in the prevention of caries, i.e., application of fissure sealants (5), caries risk documentation and preventive interventions (6), and treatment and management of dental caries (7).

For example, in one study, we used process-of-care measures and found that although 94% of patients received a caries risk assessment, the delivery of preventive therapies, including fluorides and antimicrobials, was inconsistent, with rates ranging from 56% to 94% (6). A subsequent study (7) using outcomes of care measures showed that 44% and 77% of patients had untreated caries at six months, and 20% to 35% developed new caries lesions (7). These wide variations highlight persistent clinical performance gaps in caries prevention and management.

**Figure 1:**
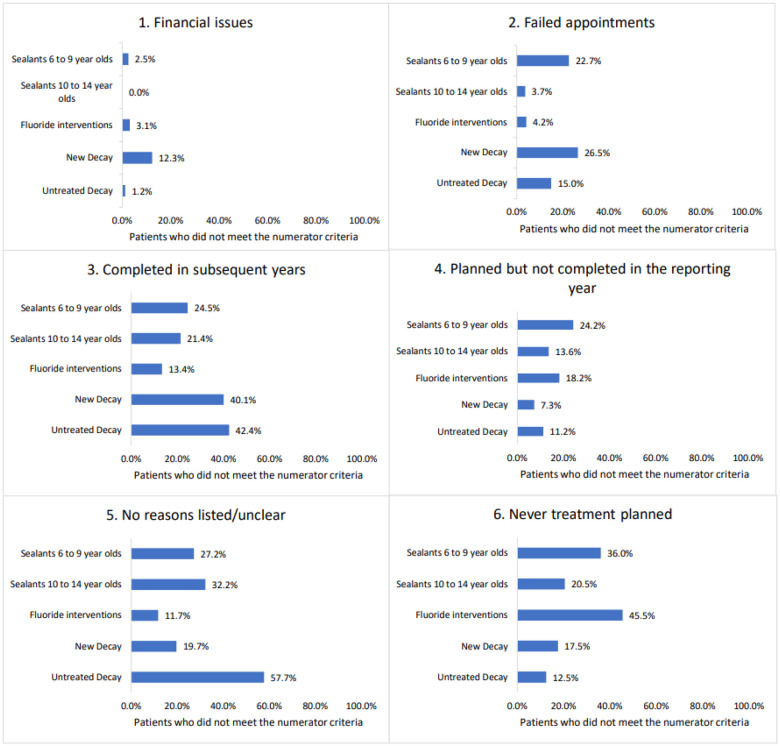
List of Contributing Factors Identified from Chart Reviews for Patients who did not meet the Quality Measure Criteria

To better understand the root causes of these quality gaps, we applied the Double Diamond (DD) Model, a structured framework with four phases: Discover, Define, Develop, and Deliver (Fig. 1) (8). The *Discover* phase (divergent thinking) involves broad exploration and understanding of the problems through data collection from various sources and perspectives related to the issue under investigation. The *Define* phase (convergent thinking) synthesizes these findings into a focused problem statement. The subsequent phase, *Develop*, focuses on generating hypotheses and potential solutions to be refined during the *Deliver* phase, which involves peer reviews, testing, implementing workflows, and refining those solutions in practice (9). In this study, we focused on the first two phases, Discover (divergent) and Define (convergent) phases, to conduct a comprehensive problem analysis of caries prevention and management across four dental institutions.

To explore the multilevel factors underlying these quality gaps, we used a triangulated, mixed-methods approach informed by principles of human-centered design and systems thinking. Our qualitative components were structured and interpreted using the AEIOU (Activities, Environments, Interactions, Objects, and Users) observational framework (10), enabling a comprehensive analysis of people, processes, tools, and environments that influence dental care delivery.

Using a multi-institutional mixed-methods study approach, we collected data through: (1) structured chart reviews, (2) semi-structured interviews with patients, caregivers, providers, and staff, and (3) ethnographic clinic observations. By triangulating these data sources, we captured what was documented, reported, and observed for a deeper understanding of institutional, electronic health record (EHR) documentation and patient-level factors that drive caries management. In this paper, we present qualitative and quantitative findings from the Discover and Define phases of the DD model to map and explain current quality gaps in caries prevention and management across clinical settings.

## METHODS

The study was conducted across four large dental care institutions: two dental schools, one large group dental practice, and one dental accountable care organization (ACO). Three of the institutions used the axiUm electronic health record (EHR) system (Exan Corp, Coquitlam, BC, Canada), while one used Epic Wisdom (Epic Systems Corporation, Verona, WI, USA). Institutional review board approval was obtained at all four sites. Guided by the Discover and Define phases of the Double Diamond (DD) Model (8, 9) (Fig. 1), we employed a triangulated, mixed-methods design to identify and understand quality gaps in caries prevention and management.

Three primary data sources were integrated: (A) Dental Quality Measures (DQMs) and structured chart reviews, (B) semi-structured interviews with patients, caregivers, providers, and staff, and (C) ethnographic clinical observations.

### A. DQMs and structured chart reviews

Across the four participating dental institutions, we implemented five DQMs, all previously validated, focused on sealants (5), caries risk documentation and prevention (6), and timely treatment of dental caries and the development of new caries lesions from our dental quality measures research (7) (Table 1). DQM scores were calculated using R (version 4.2.0) following the standardized logic detailed in (5–7). Structured Query Language (SQL) scripts were developed and tailored to each institution’s EHR system to extract relevant patient-level data. We queried encounters between January 1, 2019, and December 31, 2023. Patients who did not meet the numerator criteria for each measure were flagged as experiencing a potential gap in quality of care. Yet, results from the automated queries could not fully explain why patients did not meet the numerator criteria. For example, a patient may not appear in the numerator due to clinical documentation issues, patient-specific contraindications, clinical workflow interruptions, or patient preferences. Therefore, two calibrated independent reviewers at each participating institution conducted structured chart reviews (n = 2000) following a standardized set of Root Cause Analysis (RCA) based questions (11). Using SQL, we generated a list of 100 charts per quality measure at each site (n = 2000). The reviewers were researchers with experience in clinical dentistry, dental hygiene, quality measures research and implementation, and dental informatics. Responses from the chart reviews were entered into REDCap (12) and, after independent coding by two reviewers, were classified into six groups (1) financial issues, (2) failed appointments, (3) completed in subsequent years, (4) planned but not completed in the reporting year, (5) no reasons listed/unclear, and (6) never treatment planned.

### B. Patient, Provider, and Staff Semi-Structured Interviews

Interview guides were developed for three stakeholder groups: dental providers (e.g., dentists, dental hygienists, dental therapists), clinical staff (e.g., dental assistants, patient care advocates), and patients or caregivers (parents of patients). Each guide was semi-structured and organized into thematic sections aligned with the participant’s role and experience. Core domains included caries risk assessment, caries prevention and management, quality of care, communication practices, workflows, and barriers or facilitators to care delivery. While several domains were consistent across groups (e.g., perspectives on prevention), others were role-specific (e.g., documentation and team responsibilities for staff; at-home oral hygiene for patients). The full set of interview questions is presented in Appendix A, organized by the respondent group.

Between February 2023 and September 2023, we completed a total of 102 individual interviews across our four clinical sites: 39 with dental providers, 35 with patients or parents, and 28 with clinical or administrative staff. All interviews were conducted virtually, with some participants opting for telephone, and each session lasted approximately 40 to 60 minutes. Interviews were facilitated by members of a dedicated research team whose interviewers are experienced clinicians and clinic leaders, ensuring both subject-matter expertise and familiarity with day-to-day operational workflows.

All interviews were recorded, transcribed, and analyzed thematically using ATLAS.ti software (version 23.2.1, 2023). Three reviewers independently performed initial inductive coding, followed by collaborative review sessions to assess variations, establish calibration, and develop a primary set of codes. Following this, the reviewers continued independent coding, review, and consensus to revise existing codes, incorporate additional codes, and create code groups until the codebook was finalized. Thematic analysis was conducted based on prevalence, significance, and co-occurrence of codes and code groups. Interview data informed the design of the clinical observations that followed. All interview guides used in this study are provided in Appendix A.

### C. Clinic Observations

To complement the interviews and chart reviews, we conducted targeted clinic observations using ethnographic methods to understand real-time workflows, communication, and environmental factors influencing caries risk assessment, prevention, and management for both children and adults. These observations were conducted across all four institutions during comprehensive (D0150) and periodic (D0120) oral evaluations (13).

At each site, four observation days were completed, with each session lasting approximately three hours and clinical shifts in the mornings and afternoons (n = 64 observation sessions). Observational sessions were carried out by a multidisciplinary research team of clinicians and researchers, whose combined expertise allowed them to observe and document practice-level behaviors and workflows. Observations were guided by the AEIOU framework (described below) to ensure a structured and holistic view of the clinical work system. This framework helped categorize observations related to risk assessment, preventive interventions, procedure documentation, and treatment planning. The framework also allowed us to identify not only what occurred during dental care treatments but also how and why system-level misalignments impacted performance.

Specifically:

- *Activities* included patient check-in, medical and dental history intake, caries risk assessments (CRA), and treatment planning discussions. We observed how these activities unfolded in real time and how providers engaged with documentation tasks and CRA protocols.
- *Environment* assessments focused on physical space and design, including waiting area layout, operatory space configuration, lighting, and noise levels. We noted environmental conditions that facilitated or impeded workflow, privacy, and communication clarity—particularly at check-in desks and during patient-provider consultations.
- *Interactions* captured both verbal and non-verbal communication among users. This included greetings and directions provided by front-desk staff, coordination between dental team members (e.g., dentists, assistants, hygienists), and clinical interactions with patients and caregivers.
- *Objects* referred to tools and technologies used during care delivery. These included check-in kiosks, EHR systems, CRA forms and recommendation templates, dental chairs, instruments, and monitors. Observations noted the usability of these tools and how they supported or hindered task completion.
- *Users* included all people involved in the care experience: patients, caregivers, patient-care advocates, providers, and support staff. Their behaviors, roles, and workflows were observed to understand how responsibilities were distributed and how user interactions impacted caries risk documentation and management.

Findings from these observations were synthesized into journey maps (Fig. 3) to visualize care delivery processes, highlight breakdowns, and identify opportunities for system-level improvement.

## RESULTS

We conducted a mixed-methods study across four large dental institutions using EHR queries, manual chart reviews, semi-structured interviews with patients, caregivers, and providers, and ethnographic clinical observations. This approach, guided by the Discover and Define phases of the Double Diamond design framework (Fig. 1), revealed substantial variability in the implementation and performance of five caries-related dental quality measures related to Sealants (5), Caries Risk Assessment and Appropriateness of Care (6), and Occurrence and timely treatment of dental caries (7). Below, we present triangulated findings for each dental quality measure, highlighting challenges and opportunities to improve caries prevention and management.

### A. Sealants

#### Quantitative Findings

**Figure 2:**
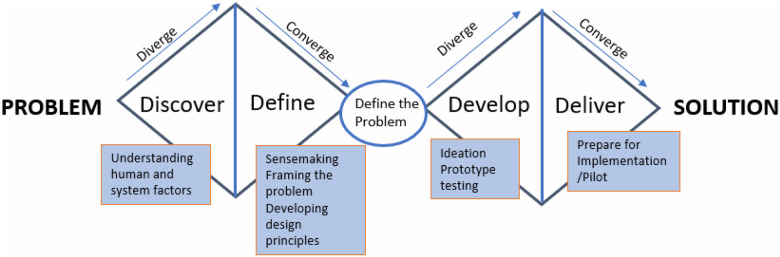
The Double Diamond Design Framework for Identifying and Addressing Quality Gaps in Caries Prevention and Management Modified from: https://www.designcouncil.org.uk/our-resources/the-double-diamond/

**Figure 3:**
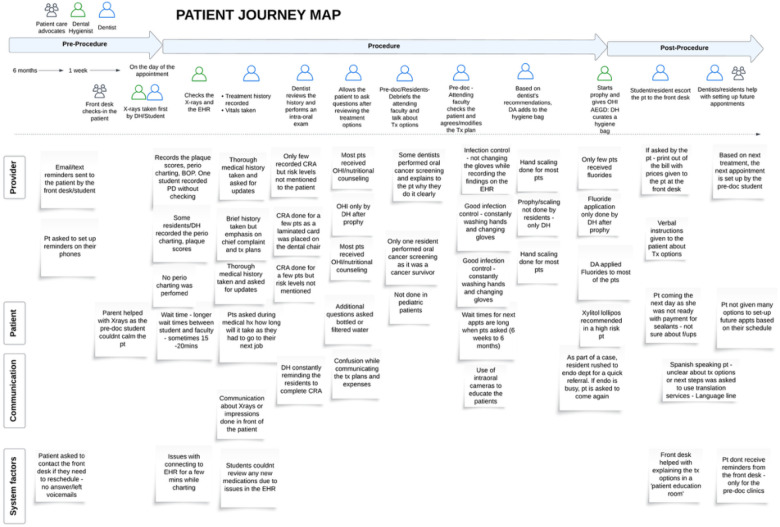
Example of a Journey Map Analysis

Between 2019 and 2023, automated EHR queries revealed notable variation in sealant placement rates across institutions: 30.3%–54.6% for first molars (ages 6–9) and 17.5%–43.0% for second molars (ages 10–14). Manual chart reviews (n = 400) found that among eligible children aged 6–9, the most common reason for not receiving sealants was *never treatment planned* (36%). Among children aged 10–14, the most frequent issue was *no reason listed/unclear* (32%). Additionally, 24% of younger children and 14% of older children had sealant treatment planned but not completed (Fig. 2).

#### Qualitative Findings

Interviews and observations indicated underuse of sealants among high-risk children and occasional overuse among low-risk patients. Barriers included workflow inefficiencies, gaps in insurance coverage, and logistical constraints with same-day sealant application. Student providers often lacked confidence in sealant placement and post-operative care, particularly in high-volume or fast-paced clinical environments.

Training variation and unclear supervision protocols contributed to inconsistent application across institutions. Participants described frustration with missed preventive opportunities due to insurance and clinical workflow constraints:

“Unfortunately, some of the sealants, especially by the students, sometimes don’t last. Then they come out. Then we get to reseal again…”

– Provider

“Insurance does not even cover sealants for adults… If I did a filling on that patient, it would be covered. But the sealant… the patient pays out of pocket.”

– Provider

“If I can do a sealant and save a cavity, I’m going to do it… If there’s a tooth that’s not sealed and they’re moderate to high, it’s getting sealed.”

– Provider

(See Table for additional participant quotes.)

These findings highlight systemic misalignments in sealant delivery, including financial disincentives for prevention, inconsistent provider training, and fragmented communication about caries risk and eligibility during patient visits.

### B. Caries Risk Assessment and Appropriateness of Care

#### B1. Caries Risk Assessment

##### Quantitative Findings

Documentation of caries risk assessment (CRA) varied widely, ranging from 50.4% to 99.6% across institutions. Among patients identified as high risk, fluoride-based interventions—used as a proxy for appropriateness of care—were delivered to 61.1%–99.0%. Manual chart reviews revealed frequent omissions in treatment planning, documentation only in free-text notes, or institutional emphasis on restorative over preventive care. Caries incidence among patients who did not receive appropriate interventions ranged from 6% to 23% (Fig. 2).

##### Qualitative Findings

Interviews and observations highlighted substantial variability in CRA implementation. CRA forms were often adapted from validated tools but differed significantly across EHR systems and lacked algorithmic support for automatic risk assignment. Usability issues led many providers, especially in satellite clinics and postgraduate programs, to underuse or skip CRA forms altogether.

CRA was commonly viewed as a student training requirement rather than a clinical decision-making tool. At some institutions, confusion existed about whether providers or hygienists were responsible for risk assessment and documentation. Preventive CDT codes (D0601–D0603) were inconsistently used, and workflow constraints frequently deprioritized CRA completion.

#### B2. Appropriateness of Care: Fluoride, SDF, and Chlorhexidine

Manual chart reviews and interviews identified multiple reasons for gaps in fluoride delivery: lack of treatment planning, incomplete documentation, or prioritization of restorative procedures. Cost was the most cited barrier for patients who declined fluoride treatments, especially when services were not covered by insurance.

Silver diamine fluoride (SDF) was used selectively due to concerns about tooth discoloration, taste, and insurance barriers. One institution excluded it entirely from their clinical guidelines. Chlorhexidine was underutilized due to age restrictions, side effects, and limited provider awareness. Hygienists and assistants were often responsible for education and preventive applications but faced unclear protocols and time constraints.

“I actually don’t know where the CRA form is in our software… if it were more accessible, we’d probably use it more.”

– Provider

“Sometimes patients won’t get fluoride because they can’t afford it—it’s $50 out of pocket.”

– Provider

“If I don’t see it in the treatment plan, I don’t bring it up. But it doesn’t mean the patient doesn’t need it.”

– Assistant

(See Table 2 for additional participant quotes.)

### C. Occurrence and timely treatment of dental caries (7).

#### Quantitative Findings

According to the EHR queries, the proportion of patients without untreated decay six months after their initial evaluation ranged from 23.1% to 56.4% across the four institutions. No New decay incidence ranged from 65.9% to 80.6%, indicating the burden of disease recurrence or progression. The manual chart reviews revealed frequent misclassification of caries risk levels, with some high-risk patients incorrectly assigned to lower risk categories, affecting up to 10% of patients at some sites. As a result, many high-risk patients did not receive recommended preventive treatments, such as prescription-strength fluoride toothpaste or chlorhexidine mouthwash, with coverage rates ranging from just 11% to 76%. In-office preventive recommendations—including nutritional counseling (CDT D1310) and oral hygiene instructions (OHI; CDT D1330)—were also inconsistently documented. In some institutions, up to 90% of high-risk patients lacked documentation of either service.

#### Qualitative Findings

Patient interviews revealed general confusion around basic oral hygiene practices, including toothbrush and toothpaste selection, flossing frequency, and appropriate use of mouthwash. These findings were consistent with observational data, which identified multiple barriers to compliance with traditional oral hygiene preventive protocols, such as low oral health literacy and competing life demands. Such contextual factors often went undocumented in the patient EHR charts but had a direct impact on patient compliance and clinical outcomes.

“I wish they had told me exactly what kind of toothpaste to buy. I didn’t know prescription toothpaste was even a thing.”

– Patient

“We give the instructions, but if it’s not in the treatment plan, it doesn’t get followed up.”

– Provider

“Some of my patients don’t floss because they don’t know how—others because they don’t think it matters.”

– Hygienist

(See Table 2 for additional participant quotes.)

#### Systems-Level Insights

Our results suggest a complex interplay between the clinical environment, EHR tools, and team communication. For example, lack of designated spaces for private counseling, limited access to educational materials, and unclear handoffs between providers and hygienists contributed to breakdowns in preventive care delivery. These findings emphasize that quality gaps in caries prevention also include system issues and are not solely behavioral or clinically driven.

#### Cross-Cutting Themes Across Measures

Several recurring themes emerged across all five quality measures. Inconsistent documentation, particularly around CRA coding, fluoride delivery, and preventive counseling, was widespread. Role ambiguity among providers, hygienists, and assistants led to gaps in care delivery and communication. Workflow inefficiencies and EHR usability issues limited the consistent application of preventive protocols. From the patient’s perspective, financial barriers and limited oral health literacy influenced treatment acceptance. These patterns were further illustrated through participant quotes presented in Table 2, highlighting how both system-based and human factors impact the reliability and consistency of caries prevention methods.

## DISCUSSION

This multi-institutional mixed-methods research study systematically mapped quality gaps in caries prevention and management across four large U.S. dental institutions. Substantial variability in sealant placement, fluoride delivery, caries risk documentation, and caries outcomes was observed, driven by intersecting system-, provider-, and patient-level factors. Inconsistent documentation, particularly around CRA codes, preventive CDT codes, and counseling, interfered with actual care delivery and limited the applicability of dental quality metrics, consistent with other healthcare fields (14).

Role ambiguity among providers, hygienists, and assistants created uncertainty around task ownership, especially regarding CRA completion and patient education. Workflow bottlenecks, time constraints, and cumbersome EHR interfaces further undermined protocol adherence. On the patient side, financial barriers and limited oral health literacy shaped treatment acceptance and follow-through. These themes, illustrated in Table 2, suggest that quality gaps are mostly system-based rather than isolated incidents of noncompliance.

Our findings are consistent with national data showing that sealant placement rates remain below public health goals (1, 5). We also found that CRA was commonly perceived as an “academic requirement” primarily for students, a finding consistently reported in studies from other academic settings (15, 16). Despite institutional policies supporting risk-based dental care, CRA completion was often bypassed due to usability barriers or deprioritized in favor of restorative procedures. This reinforces prior evidence that risk assessment, though clinically endorsed, is inconsistently implemented in real-world settings (17).

The use of the Double Diamond design framework helped us distinguish between problem discovery—quantitative identification of quality gaps—and problem definition—qualitative exploration of their underlying causes (8, 9).

Findings from this study suggest several practical implications. First, integrating decision support tools into EHRs—such as auto-populated CRA forms and clinical reminders tied to caries risk—could improve consistency in preventive care delivery. Second, clarifying standing orders so that hygienists and assistants can complete CRA, apply fluoride varnish, and document patient education may enhance role clarity and reduce missed opportunities. Third, expanding insurance reimbursement to cover sealants and fluoride across all ages, and aligning incentives with documentation completeness, could mitigate the financial disincentives frequently cited by both patients and providers. Fourth, targeted education tailored to provider roles—including students, residents, and clinical staff—may address confidence gaps and reinforce evidence-based protocols. Finally, the development of culturally and literacy-appropriate communication aids, combined with techniques like teach-back, may help overcome the patient-level barriers identified through interviews and observations.

This study has several strengths, including the integration of validated dental quality measures, triangulation of four data sources, and the use of human-centered design and systems frameworks to interpret findings. However, there are limitations. Manual chart reviews, while structured, are subject to abstraction bias. Institutional customization of EHR systems may affect generalizability. The crosssectional nature of interviews and observations may not fully capture dynamic or seasonal shifts in workflow. The presence of observers may have altered clinician behavior, although this potential Hawthorne effect was mitigated through corroboration with EHR and interview data (18).

Future research should focus on prospective trials evaluating the impact of integrated decision-support tools and team-based care models on preventive quality measure performance. Economic evaluations are needed to quantify cost savings associated with reduced restorative burden from improved prevention. In parallel, co-design efforts with patients and families could inform the development of communication tools that are contextually relevant and health-literate.

## CONCLUSIONS

This mixed-methods study advances dental quality measure research by providing a comprehensive, multi-level view of the operational and contextual drivers behind persistent quality gaps in caries prevention and management across distinct dental institutions. Through the innovative application of the Double Diamond framework and triangulated methods integrating chart reviews, interviews, and clinical observations, we identified systems-level barriers that contribute to inconsistent delivery of evidence-based preventive care.

Findings underscore the need for coordinated interdisciplinary efforts to address these gaps. Stakeholders, including dental professionals, practice leaders, policymakers, and health informatics experts, must collaborate to implement targeted solutions. Leveraging real-time data, standardized quality measures, and embedded clinical decision tools offers a promising path toward improving the consistency and effectiveness of preventive services.

This study demonstrates the value of integrating multiple data sources and user-centered approaches to inform quality improvement efforts. Such approaches can bridge the gap between evidence and routine practice, enhancing the delivery of patient-centered, preventive oral health care in real-world settings.

## Supplementary Material

This is a list of supplementary files associated with this preprint. Click to download.

- AppendixA.docx

## Figures and Tables

**Table 1:** Summary of Dental Quality Measures Used to Assess Caries Prevention and Management

Dental Quality Measure	Measure Domain	Measure Description
Sealants in 6–9 year olds (5)	Process of care	*Numerator*. The percentage of patients with elevated caries risk who received a sealant on permanent first molars in 6 to 9-year olds.
Sealants in 10–14 year olds (5)		*Denominator*. The percentage of patients with elevated caries risk who received a sealant on permanent first or second molars in 10 to 14-year-olds
Caries Risk Assessment and Appropriateness of Care (6)	Process of care	*Numerator*. The percentage of patients who received a CRA.
	Process of care	*Denominator*. The percentage of patients at elevated caries risk who received the appropriate risk-appropriate treatment.
Caries Outcome of Care - New decay (7)	Outcomes of care	*Numerator*. The percentage of patients who experienced new decay within 3 years of an exam.
Caries Outcome of Care – Untreated decay (7)	Outcomes of care	*Denominator*. The percentage of patients with an active caries diagnosis at an exam who still have untreated active decay at 6 months.

**Table 2:** Participant Quotations from Interviews Based on Caries Risk Assessment and Interventions

Dental Quality Measure	Provider/Staff/Patient Participant quotes
Sealants	*“Unfortunately, some of the sealants, especially by the students, sometimes they don’t last. Then they come out. Then we get to reseal again, so I will say that’s the thing.” (Provider)*
	*“Insurance does not even cover sealants for adults. It’s a big mess. So, if I did a filling on that patient, it would be covered. I have to drill it, actually drill it down, put a little material, and then it’s covered. But the sealant, which I don’t even have to drill, it’s going to help this patient for the next five years, if not more, hopefully longer if I do it properly and well, then I’ve saved that tooth for a longer period of time. But guess what? The patient pays out of pocket for that sealant sometimes. It could be 50 or $60” (Dental hygienist)*
	*“If I can do a sealant and save a cavity, I’m going to do it. And I do a lot of sealants. And sometimes, my assistants know when I walk in, if there’s a tooth that’s not sealed and they’re moderate to high, it’s getting sealed.” (Provider)*
Caries Risk Assessment and Appropriateness of Care	*“I think, again, it’s [caries risk assessment is] just more laborsome work that they [residents] kind of want to [not perform and] get to the good stuff on there.” (Provider)*
	*“To be honest, I actually don’t know where that is in our software. So to have it accessible and to know where it is, then we can probably utilize it a lot better.” (Provider)*
	*“But even with the recommendation, of course, some patients are very resistant of it. They could be anti-fluoride or they just don’t want it due to cost” (Provider)*
	*“I think they suggested an additional fluoride treatment, but financially, I just was like, “You know what? This is all I can spend today. I’ll just be better about what I’m doing at home” (Patient)*
	*“I think the only challenge that I would think is the cost of the toothpaste that we buy It’s getting more expensive, and insurance doesn’t cover it. And I wish they did because, in the long run it saves a lot of money. But that’s the only thing. I mean, for us, we have to make it a priority, but I can’t imagine other families that don’t have the same financial means being able to do that for multiple kids.” (Patient)*

Modified from: https://www.designcouncil.org.uk/our-resources/the-double-diamond/

## Data Availability

The datasets generated and analyzed during the current study are not publicly available due to institutional and ethical restrictions related to patient and participant privacy. The data include protected health information derived from electronic health records and interview transcripts containing potentially identifiable details. De-identified and aggregated data that support the findings of this study may be made available from the corresponding author on reasonable request and with permission from the participating institutions’ Institutional Review Boards.
